# Scarlet Fever in Horses

**Published:** 1884-04

**Authors:** John C. Peters

**Affiliations:** New York; Late President of the New York County Medical Society


### Art. IX.—SCARLET FEVER IN HORSES.

BY DR. JOHN C. PETERS, OF NEW YORK.

*Late President of the New York County Medical Society.*

Continued from page 14.

### Spontaneous Origin of Scarlet Fever.

MY first article ended with Dr. Williams and Robertson's views about the spontaneous origin of scarlet fever in horses. Williams attributes the disease to "defective drainage and ventilation of stables, by which the atmosphere becomes loaded with decomposing animal matters, which are inhaled into the lungs, become absorbed into the blood and are finally cast out upon the skin and mucous membranes, which they irritate, and where they cause eruptions. Williams, it will be seen, traces scarlet fever to external causes, viz., to impure air, from bad hygienic surroundings. Dr. Robertson on the other hand suggests a theory based upon internal causes. He says, " when horses have passed through tedious constitutional or debilitating diseases, their systems are often surcharged with effete and deleterious substances, the results of disordered or excessive tissue metamorphoses or changes ; so that an unhealthy or vitiated state of the blood is induced, which ultimately appears in the shape of scarlatinal or other eruptions upon the skin and mucous membranes. The largest portion of the medical profession are not prepared to admit any other mode of origin or propagation of true scarlet fever than contagion ; and if we admit its occurrence in horses, dogs, cats and perhaps other domestic animals, we have less need of assuming its spontaneous origin. The celebrated Dr. Copland, in his Encyclopedia of Medicine, article skin, p. 870 says, " Like small pox, scarlet fever was first generated in the lower animals, and occurred among them as a pestilence or epizooty not unlikely first among the equine race, and was then communicated to man." He also says : " I (Copland), have lately had reasons, indeed evidence, for the following inferences :

" 1st. That scarlet fever was originally a disease of the horse, and that it formerly occurred, and has even recently occurred epidemically, or as an epizooty among horses.

" 2d, That it was communicated in comparatively modern times from horses to man.

" 3d, That it may be and has been communicated also to dogs."

The earliest account of scarlet fever in horses which I have been able to find is that of Frascator in A. D. 1514. He describes a pestilential examthematic or eruptive fever among horses, which he says was a kind of quinsy, followed by eruptions, somewhat like measles, or purpuric fever in man, but which seems to me more similar to the scarlet fever of Sydenham, or possibly the malignant miliary fever of Hamilton, Alloni and Waltherius. An eruption appeared on the face, ears, neck and forelegs, attended with sore throat. Dr. George Gregory, in his well known lectures on eruptive fevers says : " We will search in vain for descriptions of scarlet fever in man, in the writings of the ancient Greeks and Romans." He places the origin of scarlet fever in man about A. D. 1610, or nearly 100 years after it was described by Frascator in horses.

In 1610, he says an epidemic angina, with a scarlet eruption raged in Spain, from which country it passed over in A. D. 1618 to Naples, then governed by a Spanish Viceroy and garrisoned by Spanish troops. He continues : "We naturally look, therefore, to Spanish and Italian authors for the first descriptions of the anginose, or malignant scarlet fever." Mercatus, A. D. 1612 ; Heredia, 1620 ; Sgambatus, 1626 ; and Cleris, 1636, are the first great writers on scarlet fever in its severer forms. It is true that a mild variety of scarlet fever, like that described by Sydenham in 1670 existed in the East at a very early date, and that small pox, measles and scarlet fever were long confounded together by the earlier physicians whose views of their genesis was precisely like those of Drs. Williams and Robertson: viz.,that they arose from impurities of the blood, either of internal or external origin, the most severe of which were supposed to break out as small pox, the next as measles, and the mildest as scarlet fever. The latter was sometimes supposed to be mere prickly heat, or Lichen tropicus, made infectious by filth and overcrowding. It is very certain that scarlet fever is of very modern origin in Scotland, for it only reached Edinburgh in 1680,when Sir Robert Sibbald, physician to Charles II of Scotland, says : "It is so recently introduced and so little understood that I cannot venture to offer any observations either on the theory of its origin or treatment." It is also true that some Arabian physicians described a species of measles, which from the extent of desquamation we may be reasonably assured was scarlet fever. It is also true that Richardson (see Diseases of Modern Life, p. 14,) has thrown out the suggestion that part of the plague of Athens was neither more or less than a terrible visitation of what we now call malignant scarlet fever, caused by the overcrowding of 400,-000 people, with their cattle, into a small walled town, in which only 50,000 persons usually lived, and the consequent filth and nastiness, aided by starvation and bad food, and the necessary poverty and impurity of the blood.

But if there is some little doubt about the spontaneous and modern origin of scarlet fever, there is less concerning syphilis. Bumstead, p. 35, 5th edition of his Treatise on Venereal Diseases, says : "Chancroid, a contagious ulcer of the genital organs, has existed in all ages, the records of which have been preserved, but there is no history of the existence of hard chancre and true syphilis prior to the year 1494. The ulcer of the genitals known to the ancients was always a local affection and never followed by secondary and tertiary symptoms; hereditary syphilis was then unknown; and finally, physicians who lived at the close of the 15th century, and who were perfectly familiar with chancroid, were struck with horror and amazement at the appearance at this time of a disease which is now known to be syphilis: they confessed that they had never seen its like before; that they were ignorant of its nature and treatment; and in their treatises upon venereal for nearly 30 years afterwards, described both diseases in separate and distinct chapters; thus showing that they did not entertain the least idea of their identity." Bumstead repeats: "According to the most reliable cotemporary authors, syphilis was first known to European nations from its appearance in A. D. 1494." Consequently it was first generated, or evolved, or imported into Europe about that time. Diphtheria also made its first appearance in Europe about A, D. 1600.

Dr. Stickler is also inclined to admit the spontaneous origin of scarlet fever in horses. He writes to me: "The more carefully I look at the various descriptions of the disease as given by various authorities, and talk with veterinarians, the more firmly convinced am I that equine scarlatina occurs as a disease of spontaneous development." The readiest historical light I can throw upon this subject is contained in the following: In A. D. 1617, Mercurialis plainly hinted that the epizooty prevailing then at Venice was the same as diphtheria and scarlet fever in man. He called it angina maligna and says: Although it originated among horses and cattle it was transmissable to man. "On account of daily heavy rains the herbage on the plains became covered with offensive and filthy mud, and the cattle eating of it became seized with irritation and putridity of the throat, and soon suffocated and died. Some when half dead with angina maligna were killed and eaten by the herdsmen and farmers, who fearing no such evil, succumbed to the noxious food. This calamity affected many herdsmen and small proprietors." I can easily produce a large number of similar instances, but the above must suffice for the present.

Sydenham believed in the spontaneous origin of scarlet fever, he writes: "Scarlet fever oftenest breaks out towards the end of summer, when it attacks whole families at once; and more especially the infant part of them. As the disease is in my mind, neither more nor less than a moderate effervescence of the blood arising from the heat of the preceeding summer, or from some other like exciting cause, I leave the blood as much as possible to its own despunation and to the elimination of the peccant materials through the pores of the skin," etc., etc.

Another allied disease of spontaneous origin is:

### Dengue, Dangay or Dandy Fever, or Scarlatina Rheumatica.

This is a peculiar febrile affection generally commencing suddenly, associated from the commencement with severe pains and swellings in the large and small joints, and followed about the third day with a curious cutaneous eruption or efflorescence, most like that of scarlet fever, beginning on the palms of the hands and spreading rapidly over the whole body, terminating in desquamation, or peeling off of the cuticle. Relapses are so numerous that some regard it as a malarious rheumatic relapsing eruptive fever, especially as it is most often caused by the great heat, filth and moisture of tropical cities. Some regarded it as rheumatic septic measles, because there were intolerance of light, red and watery eyes, etc.; others, as rhematic septic scarlatina, because the throat was sore and there were painful swellings of the glands of the neck, axilla and groins. Some thought it was a mixture of both, as the eruption was extremely variable, being sometimes smooth scarlet red and continuous as in scarlet fever; at others, rough in patches and of a darker hue, as in measles, etc. Some thought it was a septic rheumatism of a milder form than that which causes cerebro-spinal fever, etc., etc.

Rush met with it in Philadelphia in 1780, but the great Hindostan epidemic of 1824 is best known; from whence it was carried to the West India Islands and Southern United States: in fact it is said that no disease, with the exception of influenza ever had so wide a diffusion. In filthy malarious climates the evaporation after heavy rains, associated with the close and cloudy heat of sultry weather brings out all volatile poisons and ferments; in short, heat, moisture, filth and stagnation of air have always been associated with the origin and transmission of this disease.

It is well known that scarlet fever in human beings is often accompanied with so-called rheumatism. Dr. Richardson says we not infrequently meet with scarlet fever connected with acute rheumatic fever, although Golding Bird was the first to lay much stress upon it and noticed that rheumatic pains in the arms and legs are often very severe and connected as he supposed with the poisoned state of the blood which causes scarlet fever. Wilks may also be quoted to this effect.

### Surgical Scarlet Fever.

I may be permitted here to allude to surgical scarlet fever: In Reynolds system of medicine we read: "It has been doubted by some whether the scarlatini-form rash which sometimes follows operations is really scarlatinal. This eruption appears from the second to the sixth day after the operation, and in the cases which have caused the doubts is very fugitive and is the first and only symptom (there being no sore throat). But that the disease is really scarlet fever, would seem to be proved by the following observations: Ist, That it oftenest occurs in epidemics: 2d, That in a given epidemic a severe case occasionally relieves the monotonous recurrence of the very mild forms: 3d, That a precisely similar *scarlatinilla* (good word), attacks in the same epidemic, patients who have not been subjected to surgical operations and who have no open sore; and lastly by way of " veritable *experimentum crucis*, that, however freely these patients may be exposed to ordinary scarlet fever contagion afterwards, they do not contract that disease."

Ashurst, Principles and Practice of Surgery, p. 68, says: "Scarlet fever is regarded by English surgeons as a serious and not uncommon sequel of operations; but I (Ashhurst), have seen nothing in my own experience to lead me to consider its occurrence other than a coincidence. I have, however, several times seen erythema follow operations, and have known it to be mistaken for scarlet fever and erysipelas."

I do not know whether scarlet fever has often been observed after operations on horses: But in the Veterinarian, vol. 55, for 1882, p. 883 Dr. Graves reports a case of this of Purpura, or blood poisoning. A gray mare, aged four years, was fired for curb; on the 3d day there was a discharge from the nostrils, with swelling of the face and fore legs; the nose was covered with red blotches, the hair fell off, and blood exuded in places. It was thought that the mare's system was contaminated before the firing, which set up pyaemic ferment.

### Belladonna Scarlet Fever.

This opens up an entirely new field observation. All the other causes of scarlet fever have been infection, contagion, impurities of the blood, filth, etc. Belladonna and its alkaloid atropia frequently causes a diffused redness of the skin, not unlike the rash of scarlatina, but wanting in the punctated character of this specific eruption. Redness of the faces and some difficulty of swallowing render the similitude to scarlet fever very striking."

### Ptoamines.

Others have suggested the spontaneous origin of scarlet fever, to the formation of one of these substances; they present striking analogies with the vegetable alkaloids; but are alkaloids obtained from animal tissues and substances in incipient or complete putrefaction. Some are fixed, others voltatile. Some are soluble in ether, others in alcohol, and some are insoluble in both. Some respond to the tests for morphine; others give off odors resembling coniine. They are pungent to the taste, produce a sense of numbness on the tongue and a tickling in the throat. Some are actively toxic.

Thus we have passed in review almost every possible way in which scarlet fever may be generated anew, or contracted in any form.

### Purpura Haemonhogica.

This, like scarlet fever in horses is said to be usually associated with epizootics, especially of the catarrhal or rheumatic kind, notably with pink-eye.

It is decreed to be an eruptive fever of a remitting type, usually but not uniformly occurring as a sequel to some other disease. Professor Williams says the malignant form of scarlet fever is identical with purpura; while scarlatina simplex and anginosa differ from purpura. He says for a number of years he was of the opinion that all cases of purpura and scarlatina were one and the same disease; but he has had occasion to alter this opinion, as typical cases of both disorders are not uncommon in Edinburgh. The points of difference are *First*, the petechiæ in the nostrils mouth and throat, which are scarlet in color in pure scarlatina, and of a dark purple in purpura. *Second*, Sore throat is rarely absent in scarlet fever, rarely present in purpura. *Third*, Swelling of the glands is common in scarlet fever, and often followed by suppuration, which is rare in purpura. *Fourth*, The eruption on the skin in scarlet fever is marked by an elevation or standing up of the hair over the scattered spots; those of purpura, by broad even surfaces, often occupying the whole face, or an entire limb or limbs, and terminate above abruptly, as if a cord had been drawn tightly around the part. The swelling of the head is often especially great.

An excellent case of purpura, with a post mortem examination is given by Drs. Walton, Thompson, W. H. Porter, T. E. Satterthwaite, Wallace and Frey in Vol. II, No. 1, page 57 of the Journal of Comparative Medicine and Surgery.

Dr. Williams says purpura, in the great majority of instances occurs as a sequel to some debilitating disease, more particularly catarrhal fever; and its origin can be traced, in most cases to bad ventilation or drainage. When an animal suffering from influenza or catarrhal fever is kept in a well ventilated, perfectly drained, clean, good smelling and wholesome stable, and otherwise properly fed and cared for, purpura is scarcely ever seen. But if a horse both old and overworked, has been fed on poor food, and is housed in a badly drained, ill lighted, defectively ventilated and dirty stable; in fact when it is compelled to inhale the effluvia of decomposing and unhealthy urine and fæces, and its own and others bad breath, for several or many days together, its blood is so poisoned with effete products that it loses its integrity, becomes only feebly coagulated and accumulates in the weakened capillaries and smaller veins of debilitated and softened, or loosened structures, more particularly in the depending parts of the body, including the hanging down head, and thus forms those blood swellings which are so characteristic of purpura.

Again and again he has also witnessed the occurrence of pupura in horses apparently or partially recovered from influenza and strangles, which had been put to work or subjected to rather hard exercise while still debilitated, and whose capillaries and other small blood vessels were yet very delicate, soft and weak.

Professor Liautard also states that purpura is a common sequel of influenza and pink-eye epizootics, and occurs generally as an evidence of a broken down condition in horses kept at work, or put too early to work during the progress of these diseases. Purpura was the direct cause of death in a large proportion of the fatal cases.

The milder attacks in previously well conditioned horses may begin to improve in four to six days; but others take a much longer time, as a remittent fever, from slight absorption of decomposed blood, may set in, and the animal become liable to exacerbations from apparently trivial causes.

In human subjects the milder cases occur from simple thinness, softness, delicacy and fragility of the capillaries coupled with a loss of fibrin and coagulabilty of the blood. They may also be based upon a paresis of the sympathetic nervous system, as Simon has caused purpuric haemorrhages by destroying several of the great sympathetic ganglia in the neck. The capillaries and neighboring tissues were softened.

Severe cases of purpura in human beings often arise in persons whose vigor has been reduced by meagre, bad, or innutritious food, close confinement without exercise in a foul and stagnant atmosphere; or from continued fatigue, intemperance, damp lodgings, or miasmatic influences.

Although *sea-scurvy*, from deficiency of vegetables, excess of salt food and bad air in poorly ventilated ships, is a very different thing from purpura, which is sometimes called *land-scurvy*, yet they are closely allied diseases. Many of their symptoms and results are similar, yet there are great differences owing to the variation of their causes: in both there is want of coagulability of the blood, great delicacy and fragility of the capillaries, and similar petechial spots. Land scurvy and purpura were much more frequent in the earlier and middle ages, before many vegetables were eaten, and even the curing and storing of hay was comparatively unknown, so that an excessively large number of cattle and swine had to be killed and salted every fall, and the middle and lower classes lived all the winter and spring months on salt meats. The nobility and gentry by the aid of the severe game laws were the only ones who had fresh meat, venison, wild birds, etc.

The few seemingly healthy and robust persons who contract purpura have rude health and bad blood from high and free living generally increased by an abuse of the use of spirits and beer.

Strickler observed an abundant exit of red corpuscules through the unruptured walls of the capillaries in frogs and rabbits into which he had injected strong solutions of common table salt.

Parkes noticed an excess of iron and a deficiency of the solid constituents of the blood in two cases of purpura.

Injections of Ammonia and excessive doses of Iodide of Potash have caused purpura. Gaspard, Virchow, Stich and Weber produced haemorrhages by the injection of putrid matters into the blood. Among the chemical substances which will cause purpura at times, are sulphuretted hydrogen, sulphuret and sulphide of ammonia, all of which are to be found in some filthy stables.

Klebs describes as septic or tertiary haemorrhages those dependent upon the penetration of his *microsporon* into the tissues of the small arteries and veins.

In Reynolds system of medicine article, Purpura, is a description of the so-called Irish contagious purpuric disease, in which pigs have had purpuric spots on the belly, face and nose, from which a servant girl who tended them contracted febrile purpura, and the owner was also attacked, and a herdsman died.

The severest forms of purpura are to be found in connection with many infectious and contagious diseases. In olden times true black or spotted typhus fever used to be called purpura contagiosa. Purpuric measles is better known than purpuric scarlatina, and is preceeded by catarrhal symptoms, coryza and bronchial congestion. Purpura variolosa is better known than either, and the haemorrhages from many organs which attend the severest forms of yellow fever or haemo-gastric pestilence, depend upon a similar state of the blood and capillaries which accompanies and causes the severest forms of purpura. It is not known how often these contagious varieties of purpura occurs in horses and other animals; but they may easily be more frequent than is supposed.

Rheumatic purpura is well known and cerebro-spinal meningitis or foul air rheumatism of the membrances of the brain and spinal cord, is often called spotted fever, from the great number of purpuric spots which sometimes attend it.

CONCLUSIONS.

The intercourse of human beings with domestic animals is so frequent and intimate that it seems almost impossible that some of their infectious and contagious diseases should not be intercommunicable. Fortunately, most of them are not, or at least so rarely,and with such difficulty that it is not always necessary to interdict intercourse between them. Domestic animals are subject to a whole brood of zoo-contagious diseases, which are almost incapable of multiplying in the human body: they appear to be subject to a variety of malignant and contagious distempers and plagues from which men are almost altogether exempt. Still the list of intercommunicable diseases is a large one, and one which may have to be still more extended. Scarlet-fever and measles are so frequently, nay, almost constantly prevalent in human beings, and often in such a vast number of cases that it would seem very probable that these diseases may sometimes be conveyed to animals, especially as the families of some coachmen,grooms and cattle herdsmen oftentimes live in stables and barns. Cats and dogs, of all domestic animals, are most exposed to the contagion of measles and scarlet-fever, as they are so often the pets of children, and keep up their affectionate and sociable habits when their little masters or mistresses sicken; often lying for many hours, every day in the beds with them.

It is difficult of course to detect the eruptions of scarlet-fever and measles on the skin of hairy animals, so that measles, whenever it occurs in cats and dogs, will be overlooked as a mere catarrh and influenza; while scarlet-fever will be almost certainly mistaken for a more or less severe sore throat. That scarlet-fever does often occur in cats and dogs, I have little or no doubt, and refer my readers to an article on the subject in the *New York Medical Journal* for Jan. 19th, 1884.

Horses are not so closely and continuously exposed to the contagion of scarlet-fever and measles as cats and dogs; but in view of the tenacity with which the shed epithelium, dried mucus, etc., of measle and scarlet-fever patients retain their contagion; from the fact that the poison can also be carried in the hair and especially in the woolen clothes of the parents; while the urine of persons who have already had scarlet-fever or measles contains the virus thus eliminated from their systems, after exposure to it, all furnish ways in which scarlet-fever and measles may be conveyed to stables and barns and to the animals contained in them. When the poison of scarlet-fever or measles once gets into a horse-stable or cow-barn, no one can say when it will ever get out again; or what modification it may undergo; whether for better or worse, from the peculiarities of the atmosphere which prevails in these places. From the teachings of history, from the 15th century down, there seems little or no doubt that scarlet-fever, sometimes associated with malignant angina and at others with diphtheria, has prevailed among horses especially, and sometimes among cattle:

See *Sanitarian*, April 19th and 26th, May 10th and 30th, June 28th and October 18th, 1883. To my mind the evidence is overwhelming, unless the possibility be admitted that a disease very similar to scarlet-fever in man may be generated in the ways pointed out by Professors Robertson and Williams.

As to the success of protective inoculations with equine scarlatinal virus, I have little doubt, and expect to be better prepared to answer all these questions in the space of a very few months time; probably in the next issue of this journal.

